# Advances in nanotechnology for the diagnosis and management of metabolic dysfunction-associated steatotic liver disease

**DOI:** 10.1016/j.ajps.2025.101025

**Published:** 2025-02-09

**Authors:** Fenfen Li, Ruyan Yuan, Jiamin Zhang, Bing Su, Xiaolong Qi

**Affiliations:** a School of Pharmaceutical Sciences, Zhengzhou University, Zhengzhou 450001, China; b Liver Disease Center of Integrated Traditional Chinese and Western Medicine, Department of Radiology, Zhongda Hospital, Medical School, Southeast University, Nurturing Center of Jiangsu Province for State Laboratory of AI Imaging & Interventional Radiology (Southeast University), Nanjing 210009, China; c Basic Medicine Research and Innovation Center of Ministry of Education, Zhongda Hospital, Southeast University; State Key Laboratory of Digital Medical Engineering, Nanjing 210009, China

**Keywords:** MASLD, Liver fibrosis, Theranostics, Nanoprobes, Multifunctional nanocarriers

## Abstract

Metabolic dysfunction-associated steatotic liver disease (MASLD) has a high global incidence and associated with increased lipid accumulation in hepatocytes, elevated hepatic enzyme levels, liver fibrosis, and hepatic carcinoma. Despite decades of research and significant advancements, the treatment of MASLD still faces formidable challenges. Nanoprobes for diagnostics and nanomedicine for targeted drug delivery to the liver present promising options for MASLD diagnosis and treatment, enhancing both imaging contrast and bioavailability. Here, we review recent advances in nanotechnology applied to MASLD diagnosis and treatment, specifically focusing on drug delivery systems targeting hepatocytes, hepatic stellate cells, Kupffer cells, and liver sinusoidal endothelial cells. This review aims to provide an overview of nanomedicine's potential in early MASLD diagnosis and therapeutic interventions, addressing related complications.

## Graphical abstract

**Figure:**
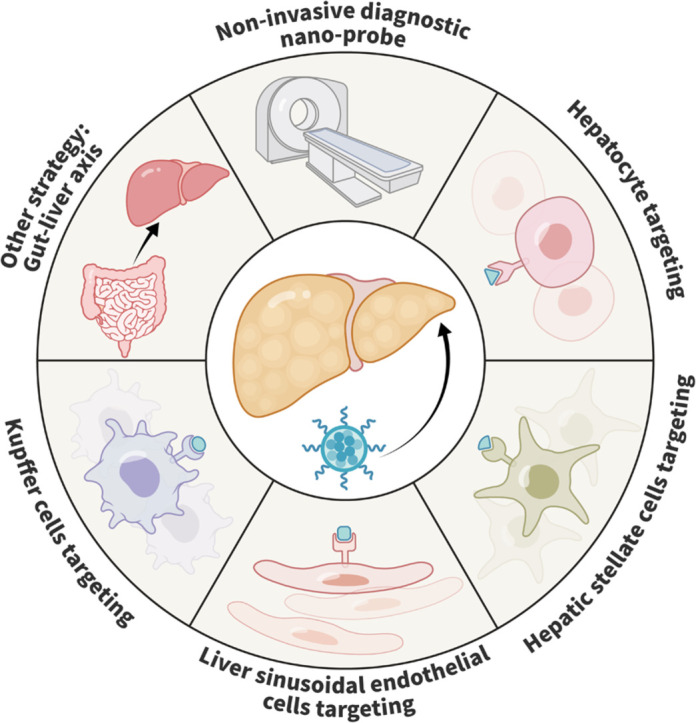

## 1 Introduction

Non-alcoholic fatty liver disease (NAFLD) is a multifactorial progressive liver disease characterized by excessive hepatic fat accumulation or steatosis, which encompasses a spectrum of pathological conditions, including simple steatosis (NAFL), non-alcoholic steatohepatitis (NASH), and fibrosis/cirrhosis [[1], [2], [3]]. NAFLD has been renamed metabolic dysfunction-associated steatotic liver disease (MASLD) [4]. According to a systematic review and meta-analysis, the global prevalence of MASLD from 2016 to 2019 was 38.0 %, representing an increase from 25.3 % during the period from 1990 to 2006 [5]. The increased prevalence of MASLD has affected the prevalence of cirrhosis. Modeling studies predict that twice as many people will suffer from MASLD-related cirrhosis in 2030 compared to 2016 [5]. Furthermore, MASLD-related complications such as cirrhosis and hepatocellular carcinoma (HCC) are on the rise, with increased morbidity and risk of all-cause mortality as fibrosis progresses [6,7]. Therefore, early diagnosis and screening for fibrosis and steatosis will benefit patients [8].

Current diagnostic methods for MASLD include invasive liver biopsy and non-invasive diagnostic tools, including magnetic resonance imaging (MRI), ultrasound (US) and positron emission tomography (PET) [9]. While liver biopsy remains the gold standard for the diagnosis of MASLD, it is limited in clinical use due to the large number of potential MASLD patients, along with sampling errors, procedural discomfort, and complications such as bleeding [[10], [11], [12]]. In contrast, non-invasive techniques like MRI, US and PET avoid the shortcomings of liver biopsy provide distinct advantages, including high operability and repeatability [13]. However, the low resolution and sensitivity of non-invasive diagnostic tools in visualizing the MASLD field make them less applicable to all patients with MASLD.

Recent advancements in nanoparticles (NPs) as intelligent probes have greatly improved the early diagnosis of MASLD and the detection of metabolic disease markers. These developments have significantly contributed to addressing this challenge. Current MASLD management strategies predominantly focus on lifestyle modification via regular exercise and eating habits. Pharmacological approaches for managing MASLD include insulin sensitizer, antioxidants, and bariatric surgery, though remain limited in scope [14]. The complex etiology, silent disease features, lack of sensitive therapeutic evaluation methods, and inefficient drug delivery systems result in few available pharmacotherapy treatments specific to MASLD patients. In 2024, the FDA approved resmetirom (Rezdiffra™), the first drug specifically for NASH with moderate to advanced fibrosis, representing a key advancement in MASLD pharmacotherapy [15]. Resmetirom can treat NASH by enhancing liver lipid metabolism. The liver-specific uptake mediated by the organic anion transport peptide 1B1 ensures its targeting to the liver. Moreover, resmetirom is a selective THR-β (thyroid hormone receptor beta) agonist, which helps avoid systemic adverse reactions associated with THR-α activation [16]. MAESTRO—NASH is an ongoing Phase 3 clinical trial to evaluate the efficacy and safety of resmetirom in humans with NASH. The MAESTRO—NASH trial currently has limitations, primarily due to the absence of clinical outcome data correlating with histological findings. Additionally, the long-term safety of resmetirom remains to be evaluated. The limited diagnostic accuracy of existing non-invasive testing criteria may lead to misclassification, potentially resulting in over- or under-treatment with resmetirom [14,15,17]. Furthermore, the FDA's conditional approval of resmetirom underlines the unmet need for improved MASLD classification and screening in primary care [18].

Recently, tremendous efforts in nanotechnology focused on enhancing early diagnostic accuracy for MASLD and addressing associated issues in lipid homeostasis, hepatic inflammation, and hepatocyte injury. The development of multifunctional probes with diverse imaging modalities makes the imaging and diagnosis of MASLD more accurate [19]. Nano-theranostic agent realize the synchronization of the diagnosis and treatment of MASLD in one integrated system [20]. Cell-specific drug delivery to the liver modulates the balance the balance between pro-inflammatory and pro-resolution signals, helping to address hepatocyte damage. Targeting Kupffer cell (KCs) inflammation also presents new therapeutic possibilities for MASLD. This review systematically summarizes recent progress in molecular targets, nanotechnology-based diagnostic tools, and treatment strategies for MASLD, providing valuable insights for researchers involved in drug discovery.

## 2 Diagnosis, pathogenesis and therapeutic targets of MASLD

Liver biopsy remains the gold standard for diagnosing, grading, and histologically assessing MASLD [21]. However, its use is debated due to associated risks, high costs, and the limited availability of effective therapies. Recently, noninvasive imaging techniques and serum biomarker-based scoring systems have been developed as alternative methods for diagnosing hepatic steatosis and fibrosis in MASLD. This section provides an overview of MASLD diagnosis, pathogenesis, and therapeutic targets.

### 2.1 Diagnosis of MASLD

The current non-invasive diagnosis of steatosis and fibrosis in MASLD, can be accomplished through imaging modalities such as ultrasonography, computed tomography (CT) and MRI, as well as the use of biochemical markers. US is the most commonly used and well-tolerated imaging tool for patients with elevated liver enzymes or for evaluating hepatic steatosis [21,22]. However, its diagnostic accuracy and sensitivity are limited, particularly for detecting mild steatosis in MASLD cases with less than 30 % fatty liver [23]. Transient elastography (TE) is an emerging non-invasive imaging technique, but in obese patients, it is associated with a failure or unreliability rate of nearly 20 % [24]. MRI-based imaging techniques are considered the most definitive tools for both qualitative and quantitative analysis of hepatic steatosis, detecting as low as 5 %–10 % liver fat content. However, high costs, long scan time, and limited availability restrict the routine use of MRI as a diagnostic tool for MASLD in clinical practice [21].

MASLD can be diagnosed using CT when liver attenuation is less than 48 Hounsfield units (HU) or when liver attenuation is lower than spleen attenuation, following the exclusion of other potential causes [21]. Qualitative assessment of the liver via contrast-enhanced CT in the portal venous phase is highly specific for detecting hepatic steatosis; however, its sensitivity remains relatively low, approximately 60 %. However, CT scans show limited accuracy in detecting mild degree hepatic steatosis and suffer from increased risk of radiation exposure, high cost, and limited availability [25,26]. In contrast, MRI demonstrates reasonable accuracy in diagnosing liver fibrosis. It utilizes higher static magnetic field strength to improve the signal-to-noise ratio (SNR). Ultrahigh magnetic fields improve sensitivity and enable sub-millimeter spatial resolution, offering novel structural and functional insights that expand its applications in clinical research. Achieving optimal depth resolution typically necessitates a powerful contrast agent (CA) to amplify the MR signal in ultra-high-field scanners, ensuring the highest possible SNR [25].

Recently, biochemical biomarkers combined with imaging biomarkers and simple parameters, including age and sex, have been investigated to predict hepatic fibrosis, the risk of NASH, and clinical outcomes [[27], [28], [29]]. The most commonly used non-invasive diagnostic and predictive tools for MASLD include serum and imaging biomarkers. Serum biomarkers include the indirect Fibrosis-4 index (FIB-4: Age × AST (IU/l) / platelet count (× 10⁹/l) × √ALT (IU/l)) and direct markers like the Enhanced Liver Fibrosis (ELF) test and NASH FibroSpect. Imaging biomarkers, such as vibration-controlled transient elastography (VCTE) and magnetic resonance elastography (MRE), are also frequently used, along with the Liver Risk score. These non-invasive tests can identify at-risk NASH patients [i.e., individuals with biopsy-proven NASH, a NAFLD activity score (NAS) ≥4, and fibrosis stage ≥2], who are candidates for pharmacologic therapy in registrational trials for treating NASH-related fibrosis [28]. Despite significant advancements, baseline differences, varied practice settings, operator dependence, and the high cost of imaging limit the broad application of these tools. Current methods still face constraints in terms of positive predictive value and generalizability across diverse patient subgroups. At present, no widely accepted, reliable, non- or minimally invasive techniques are available for differentiating simple steatosis from NASH or estimating related risk in routine clinical practice, aside from liver biopsy. Targeted probe NPs, conjugated with diverse labeling moieties and advanced CAs on a single platform, present a promising approach to meet the need for early MASLD diagnosis.

### 2.2 Pathogenesis and therapeutic targets of MASLD

**Fig. 1:**
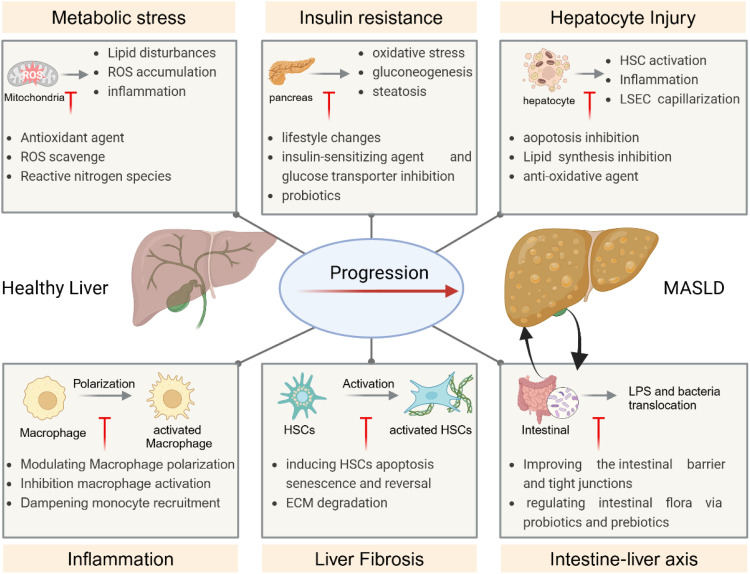
Pathogenesis and therapeutic targets of MASLD. Upon exposure to various external injurious stimuli, the liver undergoes progressive changes, eventually leading to the development of metabolic-associated fatty liver disease (MAFLD). Several factors drive this process, including metabolic stress, IR, hepatocyte injury, and inflammation. Collectively, these factors promote the activation of HSCs, resulting in excessive collagen deposition. Moreover, the gut-liver axis plays a pivotal role in disease progression. Compromise of the intestinal barrier allows the translocation of bacteria and lipopolysaccharides (LPS) into the liver, exacerbating hepatic inflammation and accelerating the progression of the disease. Created in BioRender. https://BioRender.com/o79t394.

The pathogenesis of MASLD is complex, with the initial hypothesis centering on the “two-hit theory”, which proposes lipid accumulation in liver cells as the first hit and oxidative stress injury as the second [30]. However, recent research reveals that MASLD onset is influenced by multiple factors, including insulin resistance (IR), endoplasmic reticulum stress (ER), mitochondrial dysfunction, inflammation, the gut–liver axis, among others [31]. Dysregulation within liver cells—such as hepatocytes, liver sinusoidal endothelial cells (LSECs), hepatic stellate cells (HSCs), immune cells—and their cellular interactions, along with extrahepatic influences, all play roles in the progression of MASLD [32]. Many of these pathogenic events may occur simultaneously rather than sequentially, making it challenging to precisely dissect the evolution of steatosis and inflammation. Thus, the “multiple parallel hits” hypothesis may more accurately capture the current understanding of this metabolic disease [33]. This section summarizes recent advances in elucidating the mechanisms underlying MASLD and highlights potential therapeutic targets (Fig. 1).

#### 2.2.1 Metabolic stress

In the human body, fatty acids flow continuously into the liver from multiple sources, including the hydrolysis of triglycerides (TAG) from adipose tissue, the release of dietary fat via chylomicron remnants, and fatty acids secreted by VLDL-TAG. Fatty acid metabolism occurs mainly through two pathways: esterification to form glycolipids and oxidation. An imbalance between fatty acid synthesis and utilization can result in metabolic dysregulation [34].Such an imbalance leads to lipid accumulation in the liver, an essential initial step in MASLD development [35]. Dysregulation of lipid and glucose metabolism causes metabolic stress in the liver [32]. Mitochondria and ER play essential roles in lipid metabolism disorders, reactive oxygen species (ROS) accumulation, and MASLD progression [36,37]. ER stress may activate intracellular stress pathways that exacerbate hepatic lipid disturbances, IR and inflammation. Factors contributing to hepatic lipid accumulation include increased dietary intake of free fatty acids, enhanced hepatic de novo lipogenesis (DNL), elevated adipose lipolysis, decreased β-oxidation of fatty acids, and reduced lipid transport to extrahepatic tissues [38]. Lipid accumulation impairs respiratory transport efficiency and increases ROS and ER production [37]. High ROS levels, in turn, induce oxidative damage and mitochondrial dysfunction, thereby accelerating MASLD progression [38]. Modulating antioxidative responses, particularly targeting mitochondrial superoxide (O₂*−) and hydrogen peroxide (H₂O₂), offers a promising therapeutic approach for MASLD [36,37]. NPs, such as cerium oxide NPs (CeO_2_NPs), along with antioxidant delivery systems, can attenuate oxidative stress by scavenging ROS and reactive nitrogen species, offering a novel strategy for treatment [39].

#### 2.2.2 IR

IR is a condition in which cells exhibit impaired responsiveness to insulin, disrupting glucose uptake and metabolism [38]. IR contributes to oxidative stress, inflammation, hepatocyte injury, and the progression of MASLD [40]. MASLD patients with IR show reduced insulin sensitivity across muscle, liver, and adipose tissue [41]. In skeletal muscle, decreased insulin sensitivity results in reduced glucose uptake and glycogen synthesis, leaving excess glucose available as a substrate for hepatic DNL [42]. In adipose tissue, IR is associated with an increased release of fatty acids, further exacerbating lipid accumulation and the production of TAG-derived toxic metabolites in the liver [43]. Elevated insulin levels under IR conditions promote hepatic TAG synthesis, while hepatic IR leads to glucose intolerance, uninhibited gluconeogenesis, and steatosis [44]. All these factors contribute to the excessive accumulation of lipids in the liver and MASLD development. NPs targeted delivered insulin-sensitizing drugs to hepatocytes help overcome IR with enhanced drug stability, drug concentration, and controlled release [17].

#### 2.2.3 Hepatocyte injury and inflammation

Lipids accumulate in hepatocytes and cause cytotoxicity (lipotoxicity) towards hepatocytes (ballooning) in MASLD [32]. Damage-associated molecular patterns (DAMPs) released from injured or necrotic hepatocytes activate the innate immune system and cause sterile inflammation in the liver without the involvement of pathogens and external antigens [45]. DAMPs activate inflammasomes in the livers of patients with MASLD, thereby further promoting programmed cell death, fibrosis, and cirrhosis. Nonimmune cells, such as hepatocytes and LSECs, together with innate immune cells, KCs, dendritic cells (DCs), lymphocytes and neutrophils, form a powerful inflammatory signaling pathway in MASLD [31]. Hepatic macrophages consisting of local KCs and monocyte-derived macrophages recruited from the periphery are activated and polarized towards the M1 and M2 phenotypes [38]. While M1-polarized macrophages secrete large amounts of the pro-inflammatory cytokines IL-1β, TNF-α, IL-6, nitric oxide (NO), and ROS [46]. M2-polarized macrophages exhibit anti-inflammatory effects and promote tissue repair. Modulating the macrophage balance between M1/M2 and promoting M2-induced M1 macrophage apoptosis may be a promising strategy for MASLD [46]. Inflammatory DCs are recruited to the liver and promote inflammatory responses and MASLD progression, while Treg infiltration induces the onset and progression of NASH [32].

#### 2.2.4 Liver fibrosis

The severity of liver fibrosis negatively correlates with survival in MASLD patients [38]. The degree of liver fibrosis has been identified as the primary or secondary endpoint in clinical trials for drug development targeting MASLD. Liver fibrosis is characterized by excessive extracellular matrix (ECM) accumulation in the liver due to the wound healing response and liver injury factors [47]. HSCs located in the subendothelial space of Disse are activated by inflammatory cytokines, DAMPs released by injured hepatocytes, and pathogen-associated molecular patterns (PAMPs) [17]. Activated HSCs lose intracellular vitamin A-rich lipid droplets and transform into myofibroblast-like cells that acquire proliferative and ECM-producing properties [48]. Therapeutics targeting the critical pathways involved in the dysregulation and activation of HSCs or the induction of apoptosis and senescence of HSCs are being investigated [49]. In addition, remodeling the deposition of ECM by regulating the balance between matrix metalloproteinases (MMPs) and tissue inhibitors of metalloproteinases (TIMPs) also shows therapeutic potential toward liver fibrosis in MASLD [50].

#### 2.2.5 Intestine‑liver axis

Crosstalk between the gut and liver is closely related to the development of MASLD. The intestinal barrier, which includes mechanical, chemical, immunological, and microbial barriers, allows nutrient absorption while preventing microorganisms' translocation into the lumen [51]. Disruption of the intestinal barrier decreased intracellular junction proteins, increased intestinal permeability, and disturbance of the gut microbiome are involved in the development of MASLD [32,51]. Disruption of the mechanical barrier leads to intestinal inflammation, increased intestinal permeability, increased LPS absorption into the blood, and translocation of gut bacteria, which activates KCs and contributes to MASLD progression [51]. Improving the intestinal barrier and tight junctions and regulating intestinal flora via probiotics and prebiotics are potential therapeutic targets for MASLD treatment [52,53].

## 3 Nanotechnology development in MASLD diagnosis and treatment

**Fig. 2:**
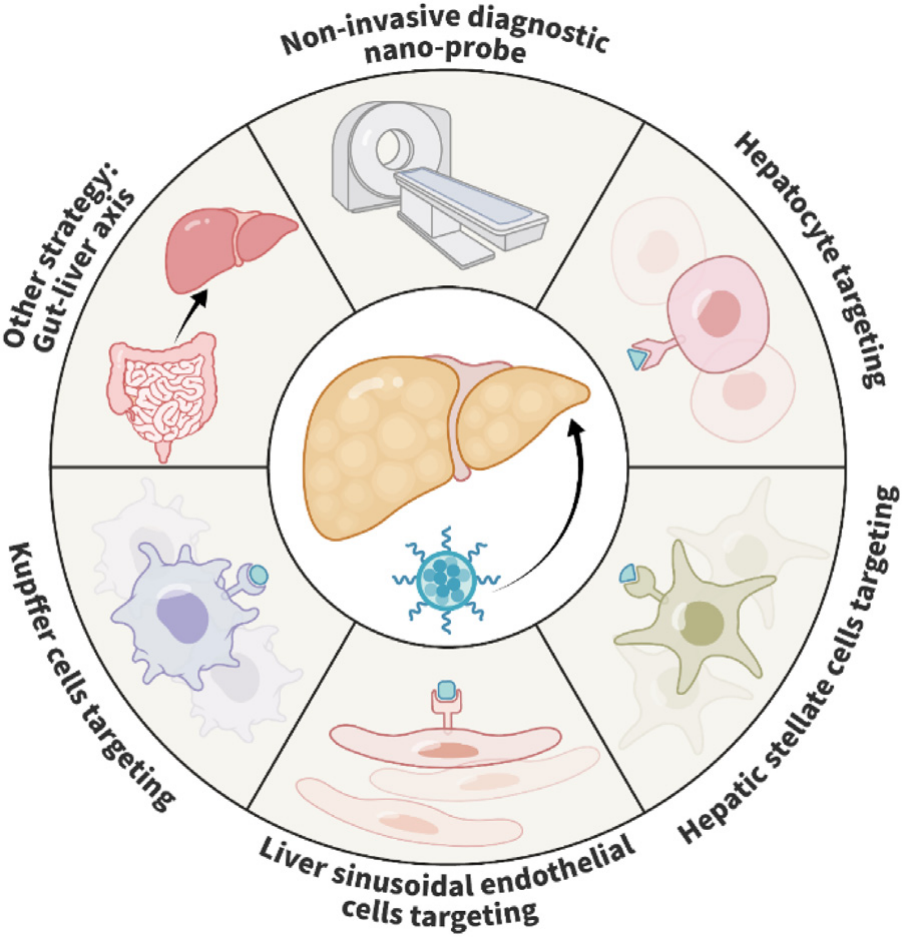
Nanotechnology-based strategies for the diagnosis and treatment of MASLD. Nanotechnology has been developed to improve the sensitivity and accuracy of non-invasive diagnosis. There are also numerous studies focused on improving the treatment of MASLD, which involves targeting hepatocytes, HSCs, LSECs, and KCs and modulating the gut-liver axis.

Current management measures for MASLD mainly include lifestyle changes, lipid-lowering agents, insulin-sensitizing medications, and diet [54]. Some promising drugs have failed to show clinical benefits for MASLD in the last stages of clinical trials [55]. A major shortcoming of these drugs is their limited concentration in the liver. Uniformly sized NPs have been developed for diagnosis and drug delivery for treating MASLD. Fig. 2 presents a summary of recent studies exploring nanotechnology-based strategies for the diagnosis and treatment of MASLD. NPs used for delivering drugs to the liver show tremendous potential for improving the bioavailability of drugs and overcoming non-specific organ toxicity [56]. Herein, we discuss the recent advances in nanotechnology for diagnosing and managing MASLD (Table 1).

**Table 1:** Delivery system for MASLD diagnosis and treatment.

	Targeting mechanism/ ligand	Imaging agent/Drug; Formulations	Mechanism	Cell lines/Preclinical models	Outcomes
Diagnosis	Endogenous	IO-DyO NPs	Improve spatial/ temporal image resolution and SNR for MRI of fibrosis [25]	HUVEC, HeLa/ CCl_4_	IO-DyO NPs contrast-enhanced T2 MR value of the liver decreases.
	Active/HA	Gd; NaGdF4@PEG@HA	PEGylated and HA modified Gadolinium based up-conversion NaGdF4 NPs targeting HSCs	Mouse HSC JS1 cells/MCD diet liver fibrosis model	Higher T1 signal for liver fibrosis diagnosis than normal ones
	Active/RGD	ICG;SPIO	Target integrin avb3 on HSCs and diagnosis of liver fibrosis [19]	LX-2, HL7702/ HF	ALT↓, good biocompatibility, long duration.
	Passive	Bi-based nanoprobes (BiF3@PDA@HA)	Elevate CT value and hold potential for precise imaging-based diagnosis of liver fibrosis [58]	HSC-T6, AML12/MCD	CT identify the dynamic changes of Bi concentration *in vivo* and generated corresponding enhancement in targeted areas on monoenergetic images, further clearly distinguishing liver fibrosis from healthy liver tissue.
	Active/β-D-Gal	GalNAc with 68Ga labeled DOTA	PET imaging diagnosis of NASH [59]	HepG2/MCD,CCl_4_	More clinically relevant than other approaches to differentiate steatosis from fibrosis, such as US-based TE.
	Active/HA	NaYF4: Gd/Yb/Er@mSiO_2_-RBS	Light controlled NO release system and NIR-II emission	CCl_4_ liver fibrosis model	Label-free imaging of liver fibrosis with high specificity and sensitivity
	Passive	Hcy-APN; Fluorescent nanoprobe Hcy-APN@MSN by self-assembling Hcy-APN and MSNs	Fluorescent probe with high sensitivity and selectivity for the detection of APN	LO_2_ cells/HFD NAFLD mouse models	Stable spectral characteristics
Hepatocytes targeting	Passive	NRG; Nanoliposome	Increase PPAR-α protein expression, correct many metabolic disturbances [63]	MCD animal model	*In vitro*: Inhibit TAG synthesis, promote TAG oxidation; *In vivo*: Improve bioavailability, oral absorption, and delivery efficiency toward hepatocytes.
	Passive	CO-releasing molecule; Styrene-maleic acid copolymer	Suppress HIF-1α expression [64]	AML12/ HFCMCD	*In vitro*: Increase the fatty acid β-oxidation and inhibit the NLRP3 inflammasome activation; *In vivo*: Ameliorates hepatic steatosis, attenuates liver fibrosis, suppresses M1 polarization and activation of the liver NLRP3 inflammasome, and restores disorders of lipid metabolism.
	Passive	CeO_2_NPs	ROS scavenger Reduce lipid peroxidation [39], decrease inflammatory cell infiltration, and attenuate inflammatory response [65].	MCD animal model	*In vivo*: Reduce the size and content of hepatocyte lipid droplets, the hepatic concentration of TG-derived MUFA, CE-derived SFA, and UFA, and messenger expression of several genes involved in cytokine, adipokine, and chemokine signaling pathways.
	Passive	FNB; ROS-responsive DSPE-PEG NPs	Reduce hepatic lipid deposition and upregulate PPAR-α [68]	MCD animal model	*In vivo*: ALT↓, AST↓, ameliorate mouse liver injury. The expression of HO-1 and PPAR-α was increased.
	Passive	IL-22 plasmid and metformin; CM	Activate STAT3/Erk1/2 and Nrf2/SOD1 signaling pathways and regulated hepatic lipid metabolism [69]	HepG2, Huh7/ HFD	*In vitro*: Enhance cellular uptake and liver-specific accumulation; *In vivo*: Alleviate hepatic steatosis, improve liver coloration and morphology, reverse liver overweight, and relieve excessive accumulation of lipid drops.
	Active/Gal	Res; OSL	Anti-lipogenesis and decrease TAG accumulation [70]	HepG2/ HFD	*In vitro*: Improve cellular uptake, decrease lipids accumulation; *In vivo*: Enhance inhibition on hepatic steatosis and IR.
	Active	Exenatide; Lipid nanocapsules	Increasing the secretion of the endogenous GLP-1 and the absorption of oral GLP-1analog [72]	RM-LNC animal model	*In vivo*: Reduce glucose levels, decrease hepatic steatosis.
	Active	Small activating RNA; PAMAM dendrimers	Upregulate HNF4A [73]	HNF4, HepG2, Hep3B, PLCPRF5	*In vitro:* Promotes metabolic regulation; *In vivo*: Regulate the factors for lipid transport (P4HB and SEC24C), lipid metabolism (YAP1), and oxidation of fatty acid (HADHB, ACADVL), reduce IL-6, liver cholesterol, liver TG, HDL/LDL and TG/HDL ratio, HNF4A↑, reduce IR.
	Passive	Self-assembling polymer-based short-chain fatty acid prodrugs	Interfering with the fatty acid oxidation pathway [75]	HFD animal model	*In vivo*: Decrease lipids accumulation and ALT.
HSC targeting	Passive	Procollagen α1(I) siRNA; C12–200 LNP	Interfere with collagen synthesis reduce collagen content [76]	CCl_4_ animal model	*In vivo*: Reduce the total collagen content in the liver.
	Active/ Vitamin A	si*HSP47*; Lipid NPs	Interfere with collagen synthesis and secretion [79]	HSCs	*In vivo*: Good tolerance, improved fibrosis biomarkers and Ishak score.
	Active/ Aminoethyl anisamide	si*IL11*; Targeted PEG-PLGA NPs	Inhibit HSCs activation, reduce liver steatosis and fibrosis [80]	HSC-T6, NIH/3T3/HFCMCD, HFHC	*In vitro*: Inhibit HSC activation, migration and invasion; *In vivo*: Inhibit HSC activation and address fibrosis and inflammation.
	Passive	Relaxin gene and miR-30a-5p mimic; Lipid NPs	Deactivate HSCs, combinatorial gene therapy achieves synergistic antifibrosis effects [81]	HSC-T6/CCl_4_	*In vitro*: Activate Nur77 receptors, KCs transfer from profibrogenic to pro-resolution phenotype; *In vivo*: Induce quiescence of the activated HSCs.
	Passive	LSKL; Self-assembling antagonist peptides	Block TGF-β activation, decrease HSCs activation [83]	CCl_4_	*In vivo*: Inhibit the deposition of collagen.
	Active/ Vitamin A	S-nitroso glutathione; POEGMA-b-VDM polymer	Release NO, inhibit collagen I and α-smooth muscle actin genes [84]	HSC-T6, LX-2/BDL	*In vitro*: Inhibition of collagen I and α-SMA; *In vivo*: Increase NO specific delivery, decrease portal pressure.
	Active/ Vitamin A	Imatinib; Liposomes	Inhibit PDGFR-β expression and reverse liver fibrosis [85]	CCl_4_ animal model	*In vitro*: Inhibit phosphorylated PDGFR-β expression, decrease heart, lung, and liver toxicity; *In vivo*: Inhibit the profibrotic pathway.
	Active/ pPB peptide	HSP 47 siRNA; Nucleic acid-lipid NPs	Silence gp46 (collagen-specific molecular chaperone) [86]	LX-2, mouse primary HSCs/TAA	*In vitro*: Increase uptake, silence gp46; *In vivo*: Increase liver distribution and HSC uptake. ALT and AST levels and the expression of α-SMA protein nearly complete normalization.
	Active/ cRGDyK	Vismodegib; Liposomes	Inhibited the hedgehog pathway and prevented the activation of HSCs [87]	Primary hepatocytes, HSCs, KCs, LSECs, biliary cells/BDL, TAA	*In vitro*: Collagen I and α-SMA↓; *In vivo*: Alleviate BDL-induced liver injury, inflammation and fibrosis, TAA-induced liver fibrosis.
	Passive	TRAIL protein, ATRA; Biomimetic PLGA NPs	Induce apoptosis of HSCs induces quiescence of activated fibroblasts [88]	LX-2/CCl_4_, MCD	*In vitro*: Induce apoptosis of the HSC; *In vivo*: Less fat accumulation, reduced hydroxyproline level, collagen I, ALT and AST↓.
KCs targeting	Passive	MMP 9 plasmid; Dendrimer−graphene nanostars	Increase MMP synthesis and secretion, promote macrophage switch to pro-regenerative phenotype [91]	RAW 264.7/CCl_4_	*In vitro*: NPs carrying MMP-9 selectively bind to pro-inflammatory macrophages, collagen I↓; *In vivo*: Reduce liver damage and improve liver recovery.
	Passive	SYK inhibitor; PLGA	Block the Fc-receptor signaling pathway and reduce immune complex-mediated inflammation [92].	RAW 264.7/MCD	*In vitro*: Inhibit M1 specific differentiation markers; *In vivo*: Suppress lipogenesis, increase lipid energy dissipation, Improve fibrosis, inflammation and steatosis.
	Active/ Mannose	Thiol groups; Polythiolated and mannosylated human serum albumin	Suppress oxidative stress and inflammation [93]	Con-A animal model	*In vivo*: Inflammatory genes level↓, improve mouse survival rate and inhibit liver fibrosis.
	Active Mannose	NO donor- S-nitrosation; Poly thiolated and mannosylated human serum albumin	Reduce oxidative stress-associated pathology [94]	KCs, RAW 264.7, J774.1 cell/STHD-01, MCD	*In vitro*: Increased uptake of Man HSA by macrophages mediated by the mannose receptor C type 2; *In vivo*: Liver protective effect.
Modulating LSECs phenotype	Passive	Simvastatin; PMs	Improving LSEC phenotype [97,105]	LSEC, KCs, hepatocytes, HSCs/BDL	*In vitro*: Reduce toxicity; *In vivo*: Increase NO specific delivery, decrease intrahepatic resistance and portal pressure.
		COX-1 siRNA; HA-PEI	Inhibit COX-1/TXA2 pathway and reduce portal pressure in cirrhotic mice [98]	LSEC, KCs/CCl_4_	*In vitro*: Increase HA-PEI/SCR targeting to LSECs; *In vivo*: Decrease the expression of TXA2, alleviate portal hypertension.
	Active/ Peptide	Riociguat+JQ1; Self-assemble peptide NPs	Restrore LSEC fenestrae and deactivate HSCs [100]	LSEC, HSCs/CCl_4,_ MCD	*In vitro*: Actively target HSC, inhibit HSC activation and collagen expression; *In vivo*: rRestore LSEC fenestrae, enhance the accumulation of IGNP-JQ1 in the Disse space, ameliorate liver fibrosis.
	Passive	Riociguat+selonsertib; Bilirubin PEG NPs	Sinusoidal Perfusion enhancement and inhibition of hepatocyte apoptosis [102]	AML12, HSC, hepatocytes/CCl_4_	*In vitro*: Inhibit the activation of ASK1 in hepatocytes, reduced hepatocyte apoptosis and HSC activation; *In vivo*: Attenuate hepatocyte apoptosis and liver fibrosis progression.
Intestine‑liver axis	Passive	Vitamin D3; Nano-structured lipid carrier	Increase in the intestinal barrier [103]	Caco-2, RAW264.7/MCD	*In vitro*: Inhibit LPS-induced macrophage activation, reduce Caco-2 monolayer permeability; *In vivo*: Reduce liver inflammation, steatosis and fibrosis.
Multi-target	Passive	Sorafenib+anti-miR155; Polyplexes	Repolarize macrophage phenotype and inhibit the proliferation of HSCs [104]	RAW264.7, HSC-T6, L02/BDL	*In vitro*: Inhibit LPS-induced expression of miR-155 in RAW264.7 cells, promote apoptosis of HSC-T6 cells, and dual targeting of KCs and HSCs; *In vivo*: Reduce pro-inflammatory cytokines in KCs, leading to inactivation of HSCs.

### 3.1 Nanoprobe for non-invasive diagnosis of MASLD

Ultra-high-field magnetic resonance imaging (UHFMRI) in clinical settings will improve resolution and diagnostic accuracy compared to conventional MRI (up to 3.0T). However, better CAs are needed to increase the MR signal in ultra-high-field scanners while achieving a satisfactory SNR [25]. A CA, specifically iron oxide/dysprosium oxide NPs (IO-DyO NPs) with a small size of 4 nm, was developed by Jiang et al. for the precise diagnosis of liver fibrosis using MRI at 7.0 and 9.4 T field strengths. Higher spatial and temporal image resolution and improved SNR have been achieved in mouse models, enabling the precise diagnosis of early liver fibrosis [25].

Unlike the abovementioned strategies, Tang et al. developed an MRI diagnostic probeGalNAc (named as NaGdF4@PEG@HA) NPs that targets HSCs for liver fibrosis imaging. Gadolinium-based up-conversion NaGdF4 NPs, serving as the nanoprobes' core, offered low toxicity and user-friendly MRI functionalization. To improve their hydrophilicity, these NPs were PEGylated, facilitating hydrogen bonding with water molecules. Additionally, the NPs' surface was modified with hyaluronic acid (HA) to selectively target CD44 receptors on activated HSCs within fibrotic liver tissue. NaGdF4@PEG@HA nanoprobe showed higher T1 signals in fibrotic livers than normal ones [57].

Further multifunctional probes with two or more diverse imaging modalities have been developed for the effective imaging of liver fibrosis. Li et al. developed a superparamagnetic iron oxide (SPIO) NP-based dual-modality probe, SPIO@SiO_2_–ICG–RGD, for the early diagnosis of liver fibrosis [19]. This NP avoided the drawbacks of easy aggregation, rapid degradation and rapid clearance from the body by preparing a novel particle that combined indocyanine green (ICG) and SPIO in aqueous solution. RGD peptide bound to integrin αvβ3 expressed on activated HSCs, and SPIO@SiO_2_-ICG simultaneously enabled imaging-guided localization of liver fibrosis through a combination of MRI and fluorescence imaging features.

It is challenging to use conventional CT in the diagnosis of liver fibrosis because of the density similarity between healthy and fibrotic livers. Incorporating nanotechnology can enhance the sensitivity of MRI and CT imaging for liver fibrosis, which is driven by activated HSCs with elevated CD44 expression. Wu et al. proposed an X-ray energy-dependent attenuation strategy with bismuth (Bi)-based nanoprobes (BiF3@PDA@HA) for the accurate diagnosis of liver fibrosis [58]. HA modified on the surface of BiF3@PDA@HA guaranteed the selective accumulation of nanoprobes in fibrotic tissues, allowing accurate imaging of fibrotic livers via spectral CT. Bi (K-edge value 90.5 keV, X-ray attenuation coefficient: 5.74 cm^2^/g at 100 keV) overcame the shortcomings of iodinated small molecules used conventionally and showed higher performance contrast enhancement on CT imaging. The surfaces of polydopamine (PDA) NPs were rich in functional groups, making them particularly suitable as a platform for building multimodal nanoCAs. In this study, PDA strongly coordinated with metal-coordinated Bi^3+^, thereby achieving effective doping of imaging components. Enhancement produced by BiF3@PDA@HA nanoprobes increased as the monochromatic energy decreased from 70 to 40 keV, expanding the scope of CT application and holding potential for precise imaging-based disease diagnosis.

Exploiting the differences in asialoglycoprotein receptor (ASGPR) expressed on hepatocytes in NASH and healthy controls, a hepatocyte-specific non-invasive probe was developed for monitoring hepatocyte function [59]. The expression of ASGPR decreases as liver fibrosis progresses. N-acetylgalactosamine (GalNAc) and 68 gallium (68Ga) labeled dodecane tetraacetic acid (DOTA) probe could detect the ASGPR level and monitor hepatocyte function in the NASH model via PET imaging, which could be further developed for early diagnosis of NASH [59].

Fluorescent probe imaging technology, used for disease detection through the identification of highly expressed active substances, boasts high sensitivity, favorable spatial and temporal resolution, and the capability of noninvasive visualization [60]. Near-infrared (NIR-II) imaging offers high spatial and temporal resolution and deeper tissue penetration. Jiang et al. designed a new therapeutic and diagnostic nanoplatform that integrated NaGdF4@PEG@HA with lanthanide nanorods and light-triggered NO release molecules (NaYF4: Gd/Yb/Er@mSiO_2_-RBS) [61]. This platform could be used for the early non-invasive diagnosis of liver fibrosis and for non-invasive monitoring of the therapeutic effects of released NO on liver fibrosis. The NP, composed of Roussin's black salt (RBS) as a photo-activated NO donor and NaYF4: Gd/Yb/Er nanorods coated with mesoporous silicon (mSiO_2_) as a carrier for gas-releasing molecules, was designed with lanthanide ions (Y/Gd/Yb/Er) doped into mSiO_2_. These nanomaterials not only displayed excellent upconversion (UC) emission signals, which laid the foundation for a light-controlled NO release system, but also demonstrated strong NIR-II emission, allowing for label-free imaging of liver fibrosis with high specificity and sensitivity.

**Fig. 3:**
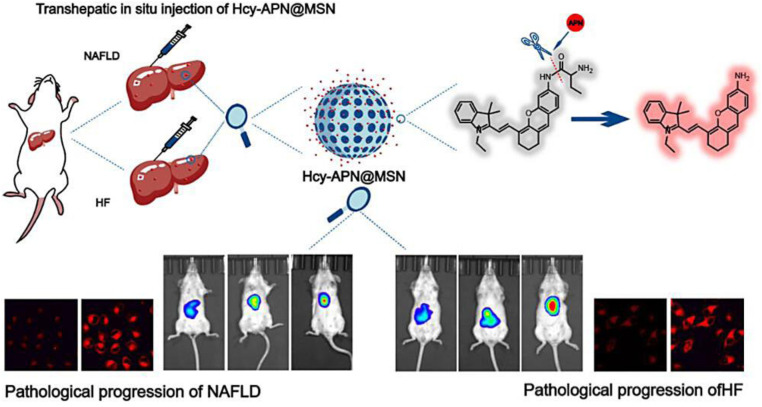
Schematic illustration showing the detection mechanism of Hcy-APN@MSN and its imaging in NAFLD mice. Reprinted with permission from [62].

Based on the abnormally high expression of the transmembrane ectoenzyme aminopeptidase N (APN) in NAFLD, Chen et al. designed a nanofluorescent probe, Hcy-APN@MSN via self-assembling Hcy-APN and mesoporous silica NPs (MSNs) in solution, for evaluating NAFLD and liver fibrosis (Fig. 3**)**. MSNs act as fluorescent nanoprobe carrier showing a high specific surface area, large pore volume, tunable pore size, and strong biocompatibility. In the liver, the hemicyanine skeleton with an amino group (Hcy) was cleaved by APN, resulting in the release of the fluorophore. Hcy-APN@MSN was employed for *in vivo* and intracellular imaging of NAFLD and hepatic fibrosis at different stages, providing more possibilities for NAFLD diagnosis [62].

### 3.2 Drug delivery systems for MASLD treatment

As described earlier, various liver cells contribute to the development of MASLD and related complications. Therefore, delivery systems targeting liver cells, such as hepatocytes, HSCs, and LSECs, have been developed to treat MASLD. Clinically, there are some problems with traditional treatment methods for liver fibrosis, such as low drug selectivity, toxic side effects on tissues and organs, and the inability to achieve effective concentrations of drugs in the liver, so the therapeutic effect is limited. Liver cells-targeted delivery systems can protect medicines from gastrointestinal effects and oral absorption and improve bioavailability, oral absorption, and hepatocyte delivery efficiency, maximizing their therapeutic efficiency. And specific delivery can effectively reduce the side effects and toxicity of liver fibrosis treatment. Here, we summarize recent advances in delivery systems targeting the treatment of MASLD.

#### 3.2.1 Hepatocytes targeting delivery system

Nanotechnology-based therapies regulate and control the multiple cellular targets involved in MASLD progression, such as NP-GalNAc-formulated small interfering RNA (siRNA), including lipid accumulation and metabolic stress. Lipid accumulation in hepatocytes and subsequent hepatocyte injury are the primary stimuli in the cascade of hepatic inflammation and ECM accumulation. Naringenin (NRG) is a hydrophobic drug with low oral bioavailability. It is encapsulated in the lipid bilayer of nanoliposomes, thereby improving the water solubility of the contained drug. Nanoliposomes encapsulating the hydrophobic drug NRG could improve bioavailability, oral absorption, and delivery efficiency targeting hepatocytes [63]. NRG nanoliposomes (NRG-Nanolipo) significantly reduced serum ALT and AST levels in MCD mice. Additionally, they inhibited lipid accumulation and demonstrated comparable effects to the crude drug at a four-fold lower dose. Recent research has shown that the hypoxia-inducible factor-1α (HIF-1α) pathway is a crucial mechanism that triggers metabolic disorders during MASLD progression. A maleic styrene-maleic acid copolymer encapsulating carbon monoxide releasing molecule (SMA/CORM2) was designed to inhibit HIF-1α expression, suppress HIF-1α mediated inflammatory cascade, and ameliorate steatohepatitis in the HF-MCD diet [64].

Nanotechnology plays a vital role in reducing metabolic stress and delaying the progression of liver fibrosis. Targeting the abnormally elevated ROS levels in MASLD, Oró et al. investigated the impact of CeO_2_NPs in CCl_4_-treated NASH rats [65]. CeO_2_NPs possess hepatoprotective activity *in vivo* against cellular damage due to their highly specific local delivery (CeO_2_NPs passively accumulate in the liver) and their ability to uptake and degrade free radicals. CeO_2_NPs significantly ameliorated hepatic steatosis and hepatic inflammatory responses in CCl_4_-treated rat liver. In a study, the investigators constructed a biomimetic nanomaterial using a macrophage membrane and encapsulating polydopamine (PDA NPs). PDA NPs delay liver fibrosis progression by successfully targeting the site of inflammation, simultaneously controlling inflammation, and eliminating ROS [66]. Poly (lactic-co-glycolic acid) (PLGA) NPs loaded with nifedipine (NFD) were developed to suppress metabolic stress and alleviate obesity-related metabolic dysfunction [67]. NFD-NPs demonstrated efficient delivery and prolonged retention of NFD in the mouse liver. Released NFD enhanced autophagic clearance through Ca^2+^/calmodulin-dependent kinase II (CaMKII) phosphorylation, reduced diet-induced IR, and improved glucose tolerance.

To achieve a synergistic inhibitory effect on lipid deposition in hepatocytes, ROS-responsive NPs loaded with fenofibrate (FNB-NP) were proposed by Du et al. [68]. The ROS-responsive peroxalate ester derived from vitamin E released FNB in response to oxidative stress and protected hepatic cells from oxidative damage. This ROS-sensitive delivery carrier enhances FNB solubility and specifically delivers FNB to fatty liver lesions. The FNB-NP formulation improved the water solubility, plasma stability, and bioavailability of FNB, significantly reduced liver lipid deposition and the ROS level, and further upregulated PPARα expression in MASLD mice.

**Fig. 4:**
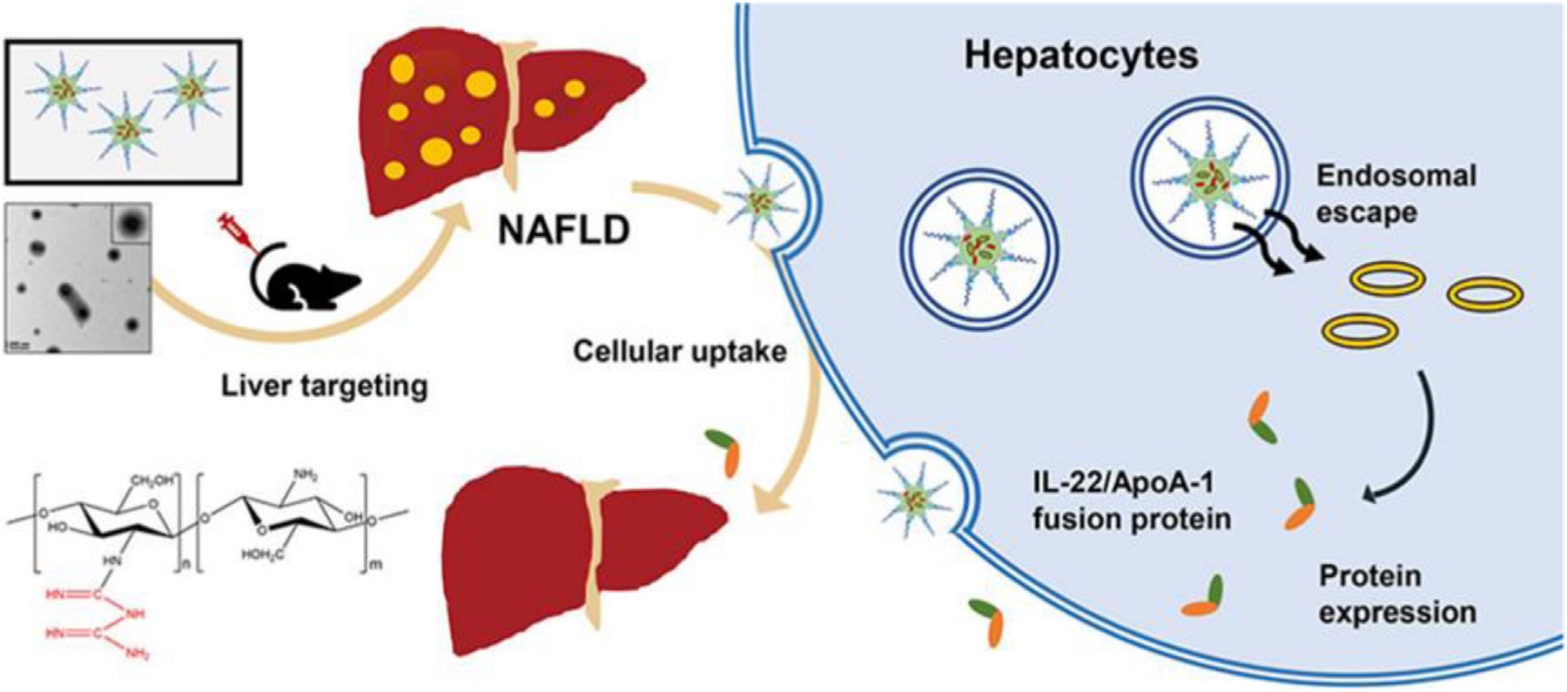
The targeted therapeutic mechanism of CDPIA NPs *in vivo*. CDPIA accumulates preferentially in the liver. Meanwhile, it achieves specific expression of the IL-22 gene and enhances its therapeutic effect on NAFLD. Reproduced with permission from [69].

Nanotechnology has made some progress in improving IR. The biguanide-conjugated chitosan in a hybrid NP carried the IL-22 and metformin prodrug simultaneously (Fig. 4) [69]. Penetratin and DSPE-PEG2000 were incorporated with chitosan–metformin (CM), and equal volumes of the CM/penetratin/DSPE-PEG2000 solution and plasmid were blended to allow the self-assembly of CM/penetratin/DSPE-PEG2000/pLA (CDPIA) complexes. The unique superiority in endosomal escape capacity and predominant liver accumulation laid the foundation for the therapeutic effects of IL-22. The delivery system alleviated hepatic steatosis, restored insulin sensitivity, and improved metabolic syndrome in an HFD mice model. This novel multifunctional gene carrier exhibits low cytotoxicity, good biocompatibility and intrinsic pharmacological activity.

Hepatic-targeted Gal-OSL/Res nanocarriers, composed of a hepatic-targeted oxidized starch-lysozyme (OSL) core, encapsulating resveratrol (Res), and functionalized with galactose (Gal) covalently conjugated on the surface, were designed for the treatment of MASLD [70]. Galactosylated ligands on the surface of nanocarriers can bind to ASGPR on hepatocytes and guarantee targeted delivery of Res to hepatocytes in the liver [70]. Gal-OSL/Res nanocarriers effectively reversed MASLD and restored hepatic insulin sensitivity in MASLD mice.

The γ-secretase complex (GSI) could ameliorate NASH and fibrosis by inhibiting aberrant Notch activity in hepatocytes; however, non-elective intestinal Notch inhibition caused goblet cell metaplasia [71]. GSI-loaded PLGA NPs targeted the delivery of γ-secretase inhibitors to the liver and reduced hepatic glucose production, hepatic fibrosis, and inflammation without apparent gastrointestinal toxicity [71]. Glucagon-like peptide-1 (GLP-1) inhibits glucagon release, slows gastric emptying, and reduces food intake. However, its rapid clearance by the circulating enzyme dipeptidyl peptidase IV (DPP-IV) limits its use. Given the above shortcomings, oral administration of lipid nanocapsules loaded with a GLP-1 analog (exenatide) was developed by Beloqui et al., which protected the drug from gastrointestinal tract digestion and showed a more substantial effect on MASLD-associated metabolic syndrome than the subcutaneous formulation [72]. Compared with subcutaneous injection of such drugs, lipid nanocapsules protected drugs and facilitated their entry into the bloodstream through the gastrointestinal tract while stimulating the release of endogenous GLP-1 and increasing its physiological levels. This dual-action strategy was proven to be effective in T2DM mouse models.

Hepatocyte nuclear factor-4 alpha (HNF4A) expression is critical for hepatocyte differentiation and glucose, fatty acid, cholesterol, and drug metabolism in the liver. The expression of HNF4A was significantly reduced in NASH and high-fat diet (HFD)-fed diabetic models. 5-(G5)-Triethanolamine-core polyamidoamine (PAMAM) dendrimers loaded with small activating RNA were developed to upregulate HNF4A in HFD rat livers, which induced a favorable metabolic profile in the liver [73]. Although short-chain fatty acids (SCFA) have been reported to alleviate NASH symptoms in mouse models. Their water-soluble nature, low molecular weight, and rapid clearance limit their clinical use [74]. Self-assembling polymer-based short-chain fatty acid prodrugs were designed to deliver SCFA to the liver. Nanoassembled prodrug (NanoSCFA) potentially improved the pharmacokinetic properties of SCFAs. It restored fatty acid β-oxidation by inducing nuclear PPARα expression in the periportal hepatocytes while also decreasing the expression level of carnitine palmitoyl-transferase 1A (CPT1A) in the hepatic tissue. The study established a suitable prodrug for NASH treatment [75].

#### 3.2.2 HSCs targeting delivery system

Stimulated by damage factors, HSCs are activated and secrete large amounts of collagen [76]. Therefore, interfering with collagen synthesis or inducing senescence, deactivation, and apoptosis of activated HSCs are promising targets for anti-fibrotic agents. Once activated, HSCs synthesize and secrete large amounts of procollagen, during which the collagen-specific chaperone heat shock protein 47 (HSP47) facilitates proper triple-helix formation and translational regulation of procollagen, leading to fibrosis [77]. The siRNA is delivered to HSCs, it can antagonize HSP47 and improve liver fibrosis. So, the critical role of HSP47 in synthesizing and screening diverse collagen types makes it an excellent candidate for modulating collagen using siRNA.

In line with this idea, Niitsu et al. tested the effect of vitamin A-conjugated liposome-encapsulated siRNA targeting gp46, the rat homolog of human HSP47, on HSCs. The surface-modified vitamin A on the NPs is a specific ligand for the RBP receptors on HSCs. The vitamin A modification on the surface of the NPs ensured their targeting and specific delivery to HSCs [77]. RBP receptor-specific liposomes VA-lip-siRNAgp46 resolved liver fibrosis progression and prolonged survival in rats with otherwise lethal dimethylnitrosamine-induced liver cirrhosis in a dose- and duration-dependent manner. Based on these encouraging preclinical data, results from a phase I clinical trial (ND-L02-s0201, a chemically modified siRNA targeting HSP47) indicated that ND-L02-s0201 was well tolerated in Japanese subjects with advanced liver fibrosis, with no dose-limiting toxicities observed up to a dose of 0.6 mg/kg over 5 week and demonstrated an acceptable pharmacokinetic profile [78]. Histological improvement was observed in all tested doses, supporting further evaluation of the efficacy and safety of ND-L02-s0201. Further phase 2 clinical trials showed that intravenous (*i.v.*) injection of BMS-986263 (a retinoid-conjugated lipid NP (LNP) containing HSP47 siRNA) resulted in improvements in METAVIR and Ishak scores and was generally well tolerated over 36 week [79]. Results from the Phase II clinical trial support further research into the role of BMS-986263 in patients with active fibrogenesis.

C12–200 cationic lipid NPs encapsulating siRNA against procollagen α1(I) were designed to interfere with collagen synthesis, specifically reducing hepatic collagen content and slowing down liver fibrosis progression [76]. Excessive IL-11 in fibrotic liver induces the activation of HSC and noncanonical extracellular signal-regulated kinase (ERK) in pro-inflammatory and fibrotic responses in liver fibrosis. Zhang et al. designed aHSC-targeted aminoethyl anisamide (AEAA) NPs encapsulating siRNA against IL11 for liver fibrosis treatment [80]. AEAA NPs loaded with *siIL11ra1* accumulated in HSCs blocked noncanonical IL-11/ERK signaling and resolved liver fibrosis in an HFCMCD-induced NASH model [80]. In another study, Hu et al. developed a lipid NP-based combinatorial gene therapy encapsulating the relaxin gene and a miR-30a-5p mimic [81]. Relaxin induced the quiescence of HSCs through exosomes released by macrophages containing miR-30a-5p. Based on these results, lipid NPs loaded with the relaxin gene and a miR-30a-5p mimic achieved synergistic antifibrotic effects with decreased infiltration of inflammatory cells in a steatohepatitis model. This study demonstrated the potential of the complementary application of nanotechnology and basic science for liver fibrosis treatment.

Transforming growth factor (TGF) is a robust profibrotic cytokine that induces HSC activation [82]. The short antagonist peptide Leu–Ser–Lys–Leu (LSKL) blocks TGF-β activation by inhibiting thrombospondin-1. However, its rapid degradation and insufficient accumulation limit its clinical application. A self-assembled nanostructure based on dipeptide Phe-Phe (FF)-modified LSKL was designed by Zou et al. to decrease collagen accumulation in the liver [83]. Through π-stacking and the hydrophobic effect between FF and LSKL, the conjugates assembled into a nanostructure, enhancing the pharmacokinetic properties of LSKL. Moreover, gaseous NO exposure benefited liver fibrosis treatment via downregulating profibrogenic TGF-β and collagen. Wang et al. proposed a vitamin A-coated NPs encapsulating NO donor S-nitrosothiols that achieved controlled and specific delivery of NO to the HSCs, thus inhibiting collagen production and decreasing portal pressure (≈20 %) [84]. The vitamin A on the surface guaranteed the specific delivery toward HSCs. In addition, Vitamin A-coated liposomes were also investigated to deliver the antifibrotic agent imatinib to HSCs in liver fibrosis treatment with decreased heart, lung, and liver toxicity [85].

**Fig. 5:**
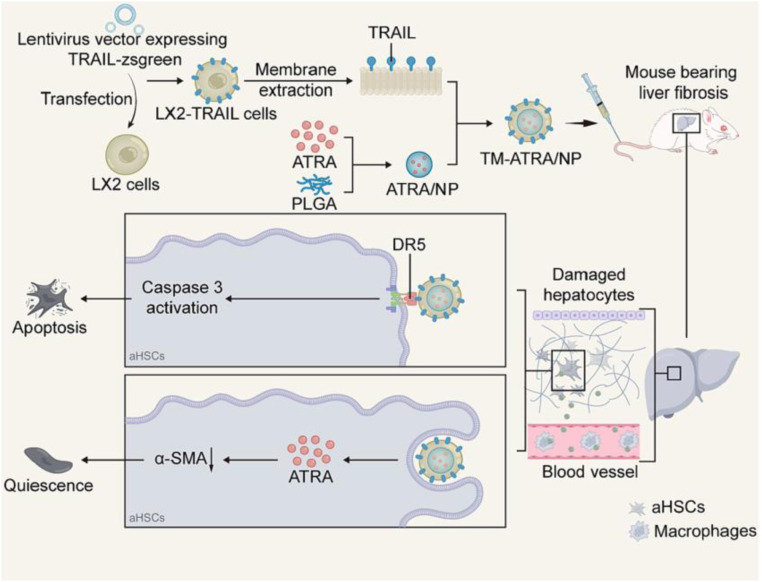
Schematic illustration showing the anti-fibrotic mechanism of TM-ATRA in liver fibrosis treatment. LX-2 cells transfected with lentiviral vector encoding TRAIL on the cell membrane. LX-2 cell membrane was extracted and coated on ATRA loaded PLGA NPs. The biomimetic nanocarrier synergetically depletes HSCs by inducing the quiescence and apoptosis of HSCs. Reproduced with permission from [88].

Taking advantage of the upregulated platelet-derived growth factor receptor β (PDGFR-β) on activated HSCs, a cyclic oligopeptide (C*SRNLIDC*)-modified stable nucleic acid-lipid delivery system was prepared to deliver HSP 47 siRNAs targeting HSCs for liver fibrosis treatment [86]. Similarly, cyclic peptide (cRGDyK) liposomes were developed to deliver the Hedgehog inhibitor vismodegib to activated HSCs and prevent activation of HSCs for liver fibrosis treatment with reduced side effects [87]. Interestingly, Liu et al. reported biomimetic PLGA NPs with tumor necrosis factor-related apoptosis-inducing ligand (TRAIL)-expressing HSC membrane coating for effective liver fibrosis treatment [88]. TRAIL-expressing HSC membranes guarantee targeted binding of HSCs and effectively induce apoptosis of HSCs (Fig. 5). The encapsulated all-trans retinoic acid (ATRA) induced quiescence of the HSCs. Ultimately, a synergistic antifibrotic effect was achieved by inducing apoptosis and quiescence in one nanocarrier in carbon tetrachloride-induced and methionine- and choline-deficient L-amino acid diet-induced liver fibrosis mouse models.

#### 3.2.3 KCs targeting delivery system

Activated KCs play a central role in inflammatory responses, HSC activation, and MASLD progression [89]. KCs act as scavengers and phagocytes in the liver, laying the foundation for liver-targeted delivery [90]. NPs with a spherical shape, positive charge, and diameter >200 nm are more easily taken up by KCs in the liver [17]. Graphene-based nanostars, with an average particle size of 237.9 nm, a zeta potential of 36.6 mV, and linked to PAMAM-G5 dendrimers were designed to selectively deliver collagenase MMP-9 plasmid to inflammatory macrophages in the fibrotic liver. Increased MMP-9 alters the imbalance between collagen synthesis and degradation, which leads to decreased collagen content [91]. Overexpression of MMP-9 also promotes the macrophage switch toward the pro-regenerative M2 phenotype.

**Fig. 6:**
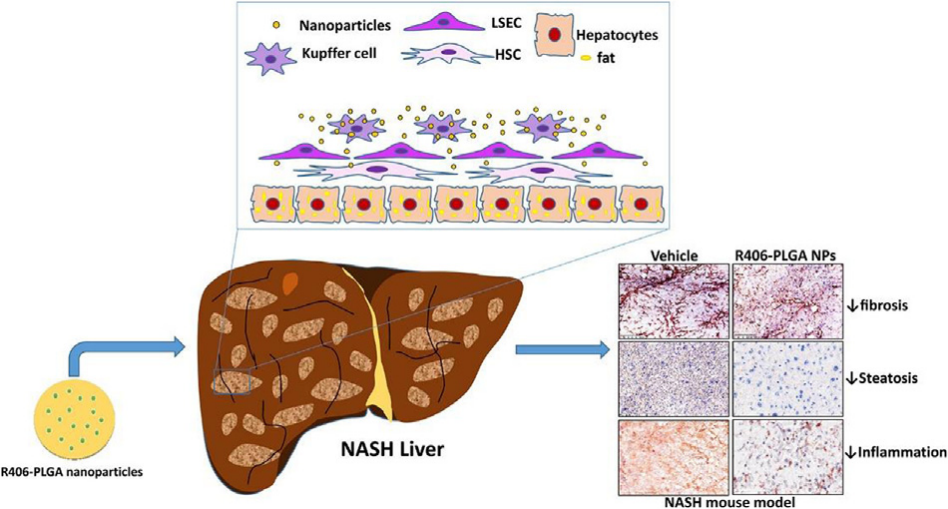
Therapeutic Mechanism of R406-PLGA in NASH livers. R406-PLGA: PLGA NPs loading SYK inhibitor R406. Reproduced with permission from [92].

The phenotype of macrophages is closely associated with the progression of NAFLD. Bansal et al. proposed that PLGA NPs modulate the inflammatory phenotype of macrophages in NASH via spleen tyrosine kinase (SYK) inhibition (Fig. 6) [92]. SYK was closely associated with disease progression, and its expression was significantly upregulated in macrophages. SYK inhibitor R406 was encapsulated in PLGA NPs. R406-PLGA further enhanced the pharmacokinetic properties of R406. R406-PLGA NPs inhibited NO release and M1-specific markers in M1-differentiated macrophages in a dose-dependent manner. PLGA NPs encapsulating the SYK inhibitor R406 significantly ameliorated inflammation, steatosis, and fibrosis in a murine model, providing a new strategy for NASH treatment [92].

In response to the overproduction of ROS by KCs, Maruyama et al. designed KCs targeting polythiolated and mannosylated human serum albumin (SH-Man-HSA) nanoantioxidants for NASH treatment [93]. In the nanoantioxidant, mannose facilitates targeted recognition and uptake by KCs, and the poly-thiol groups scavenge the overexpressed ROS, thus suppressing oxidative stress and inhibiting the inflammatory response [93]. The same group further functionalized SH-Man-HSA via S-nitrosation to increase hepatic blood flow and antioxidative activity in one nanosystem [94]. The S-nitrosation of SH-Man-HAS (SNO-Man-HSA) delivered NO and thiol groups to the liver simultaneously, which showed promising hepatoprotective effects in the NASH model.

#### 3.2.4 Delivery system modulating LSECs phenotype

LSECs, the most abundant non-parenchymal cells in the liver, play an essential role in maintaining the normal transfer of nutrients, lipids, and lipoproteins [95]. Under normal physiological conditions, LSECs exhibit anti-inflammatory and anti-fibrogenic properties by suppressing KCs and HSCs activation. However, LSEC became capillaries, contributing to the recruitment of inflammatory cells and promoting liver injury in MASLD. At the same time, LSECs also fail to maintain HSCs quiescence and promote the progression of liver fibrosis [95,96].

Considering the critical role of LSECs in intrahepatic vascular resistance and portal hypertension, Martell et al. designed a Pluronic® nanocarrier loaded with simvastatin targeting LSECs for the treatment of chronic liver disease [97]. Self-assembled polymeric micelles (PMs) based on Pluronic® amphiphilic copolymers demonstrated excellent biocompatibility and biodegradability. Simvastatin-loaded PMs showed superior effects in decreasing portal hypertension in advanced chronic liver disease, with reduced toxicity compared to free simvastatin via specific delivery targeting LSECs. The release of simvastatin in LSECs improved the cell phenotype and bioavailability of endothelial-derived vasodilator NO, thus decreasing intrahepatic resistance and portal hypertension [97].

**Fig. 7:**
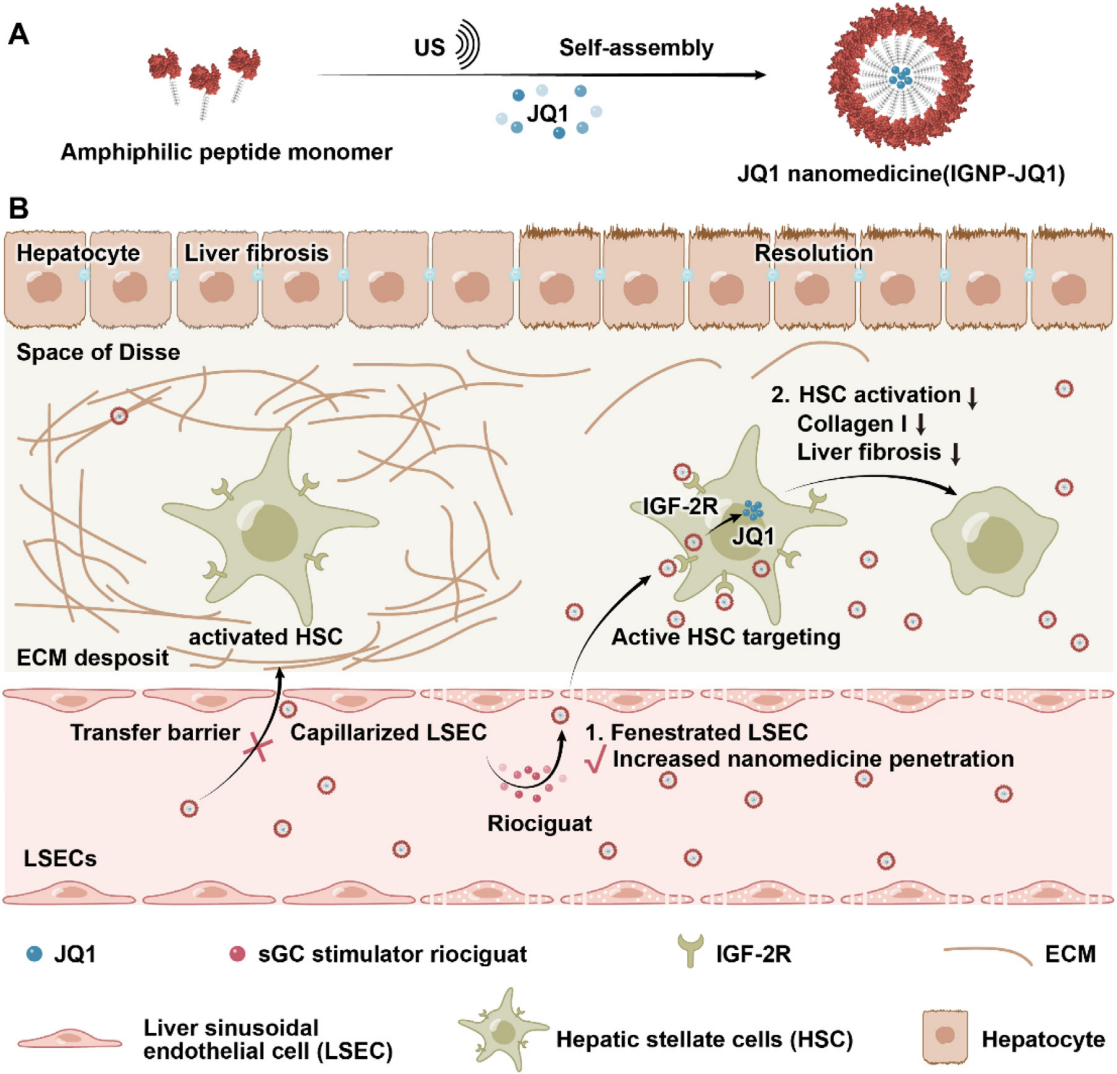
Schematic illustration showing the anti-fibrotic mechanism via LSECs phenotype restoration and HSCs collagen synthesis inhibition. Riociguat restored the porosity of LSEC via sGC stimulation, which enhanced the transport of IGNP-JQ1 into the Disse space. IGNP-JQ1 suppressed collagen and α-smooth muscle actin (α-SMA) expression in the liver of the fibrotic murine model. Reproduced (adapted) with permission from [100], Wiley. (For interpretation of the references to colour in this figure legend, the reader is referred to the web version of this article.)

LSECs also contribute to the overexpression of vasoconstrictor thromboxane A2 (TXA2) via the cyclooxygenase-1 pathway, which contributes to the onset of hepatic endothelial dysfunction and portal hypertension [98]. Hyaluronate-graft-polyethyleneimine (HA-PEI) loaded with COX-1 siRNA was developed to silence COX-1 and modulate LSECs [98]. HA-PEI mediated efficient siRNA delivery and inhibited the COX-1/TXA2 pathway in LSECs, significantly ameliorating portal pressure in cirrhotic mice. Capillarized LSECs also decreased the substrate transfer efficiency between the blood and cells in the liver, which reduced the therapeutic effect of anti-fibrotic drugs [17,99]. Our group developed a combinational strategy for liver fibrosis treatment by restoring the phenotype of LSECs and decreasing collagen synthesis in HSCs (Fig. 7) [100]. The soluble guanylate cyclase (sGC) stimulator riociguat restored the phenotype with increased porosity, which laid the foundation for the transport of HSC-targeting peptide tailor-made nanomedicine. We used a self-assembled peptide nanocarrier (IGNP-JQ1) that actively targets the insulin-like growth factor 2 receptor (IGF-2R) on HSCs to deliver the IGF-2 receptor-mediated anti-fibrotic agent JQ1 to the liver. After riociguat restored the fenestrae of liver sinusoids, the distribution of IGNP-JQ1 in HSCs significantly increased. IGNP-JQ1 decreased collagen synthesis and the number of HSCs, which reduced collagen deposition in the liver.

**Fig. 8:**
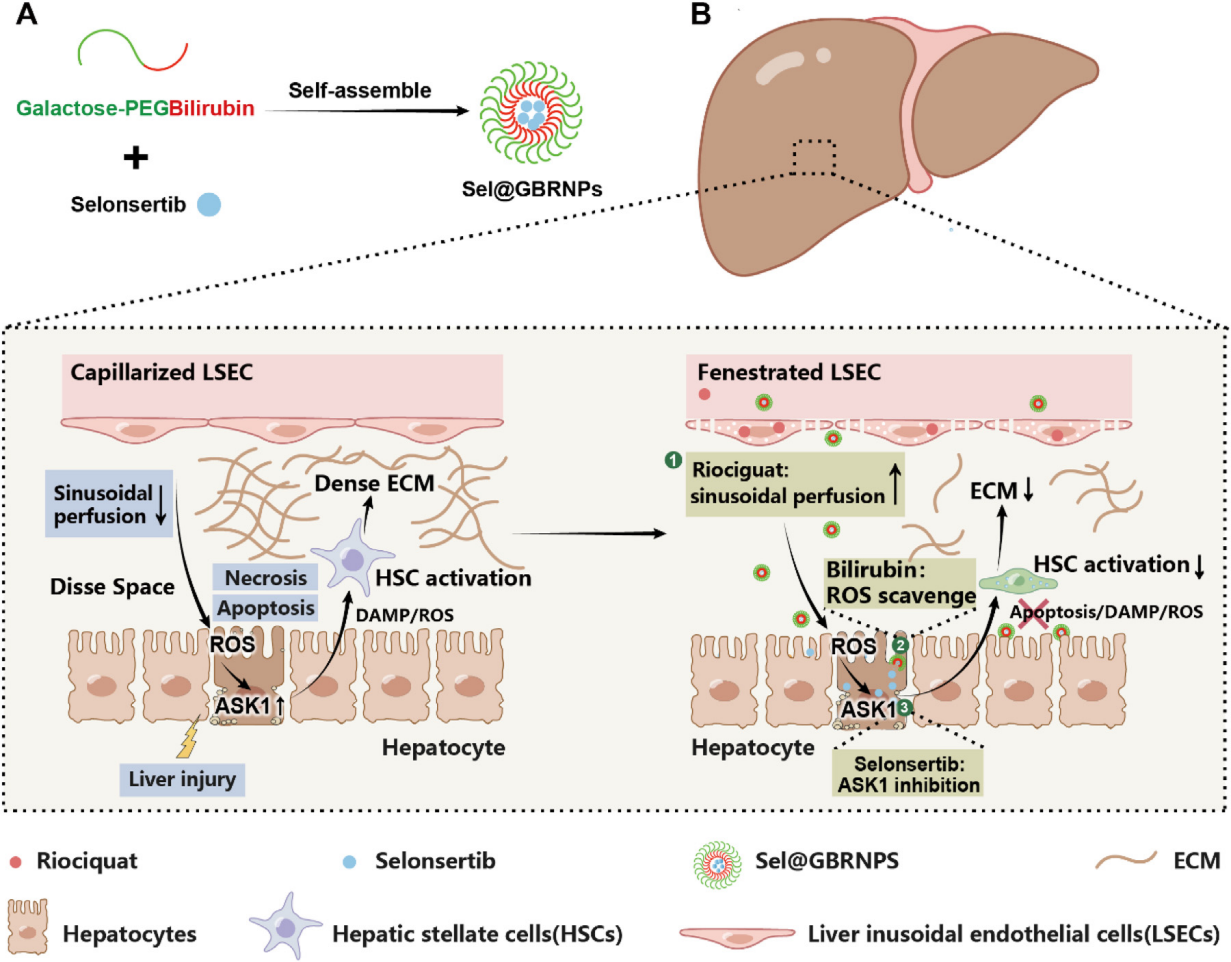
Schematic illustration of sinusoidal perfusion enhancement and hepatocyte apoptosis inhibition strategy for liver fibrosis treatment. (A) Scheme showing the synthesis of Gal-PEGylated bilirubin nanomedicine. (B) Scheme showing the proposed anti-fibrotic mechanism of riociguat and Sel@GBRNPs in fibrotic liver. Riociguat enhances the sinusoidal perfusion, enhances the sinusoidal perfusion, and ameliorates hypoxia, while Gal-PEGylated bilirubin NPs encapsulating selonsertib (Sel@GBRNPs) scavenge the excessive ROS and inhibit ASK1 activation. Decreased hepatocyte apoptosis, decreased HSC activation, and ECM are achieved via the combined modulation strategy. Reprinted (adapted) with permission from [102]. Copyright 2024 American Chemical Society.

Capillarized LSECs also caused intrahepatic vascular resistance and decreased sinusoidal perfusion in the liver, accompanied by hypoxia and increased ROS accumulation in the liver [101]. We examined the combined strategy of sinusoidal perfusion enhancement and hepatocyte apoptosis inhibition for liver fibrosis treatment (Fig. 8) [102]. First, the sGC stimulator riociguat was administered to increase sinusoidal perfusion and ameliorate hypoxia in the liver. Gal-PEGylated bilirubin nanomedicine (Sel@GBRNPs) was used to scavenge excessive ROS and inhibit hepatocyte apoptosis in the liver. The combined strategy decreased hepatocyte apoptosis and decreased stimulation toward HSC activation, which decreased ECM deposition in the fibrotic mouse model.

#### 3.2.5 Other strategy

The gut-liver axis plays a vital role in NASH progression. Decreased intestinal barrier and increased LPS PAMPs stimulation caused liver inflammation [103]. A Lipid-based nanocarrier containing active vitamin D3 (NLC) was designed to repair the intestinal barrier for NASH treatment [103]. NLC ameliorated the intestinal barrier and attenuated steatosis, inflammation, and fibrosis in the liver, with increased bioavailability compared to free active vitamin D3. Sun et al. proposed a KCs and HSCs dual-modulating chemo-gene strategy for liver fibrosis treatment [104]. Fluoropolymers with fluorine chains on the polymer backbone were synthesized to improve the affinity of polycations for cell membranes and endosome/lysosome membranes, thereby facilitating delivery efficiency for gene therapy [104]. Fluoropolymers self-assemebled NPs (Fluoro-NPs) loaded with miR155 inhibitor (anti-miR155) and sorafenib can polarize the macrophage phenotype to anti-inflammatory M2 and inhibit HSCs proliferation. This work proposes a novel strategy involving the dual regulation of KCs and HSCs for liver fibrosis treatment.

## 4 Summary and future perspectives

Given the high prevalence, diverse pathological manifestations, and scarcity of effective treatments for MASLD, there is an urgent need for the early diagnosis and the development of innovative pharmaceuticals. Nanotherapeutics emerges as a promising approach to meet the unmet medical needs of MASLD. Nanotechnologies, by enabling the targeted delivery of diagnostic probes and drugs to liver cells, can significantly enhance imaging contrast and drug bioavailability while minimizing side effects. This targeted approach is particularly crucial for MASLD, where traditional treatments may have limited efficacy and can lead to adverse effects. The precision of nanotherapeutics lies in its ability to concentrate therapeutic agents at the site of disease, thereby improving treatment outcomes and reducing systemic exposure, which is a significant step forward in personalized medicine. Advancements in nanotechnology have significantly enhanced the diagnosis of MASLD by improving imaging contrast and bioavailability in preclinical models. Nanoprobes such as IO-DyO NPs and NaGdF4@PEG@HA improve MRI contrast, offering higher spatial and temporal resolution and enhanced

SNR, which are critical for precise diagnosis of liver fibrosis. Multifunctional probes like SPIO@SiO_2_–ICG–RGD integrate MRI and fluorescence imaging, providing a dual-modality approach to enhance diagnostic accuracy. Moreover, Bi-based nanoprobes for spectral CT address the limitations of conventional CT, enabling accurate differentiation of fibrotic liver tissue. These advancements represent innovative and effective strategies for MASLD diagnosis. Nanotechnology offers promising solutions for addressing the progression of MASLD by targeting cellular processes such as lipid accumulation, inflammation, and metabolic stress, HSC activation and barrier caused by LSEC capillarization. Beyond simply loading drugs into nanocarriers, drugs can be conjugated to the polymer backbone via ester, amide, or pH-responsive bonds to enhance pharmacokinetic properties. Advances in nanotechnology have enabled the precise targeting of liver cells and the underlying processes driving disease progression. This breakthrough offers hope for more effective treatments with significantly reduced side effects. Despite encouraging results and the advantages of nanocarriers, only one targeted lipid NP delivery system has advanced to phase 2 clinical trials. This system, BMS-986263, delivers HSP47 siRNA to HSCs (NCT03420768) and has shown histological improvement in METAVIR and Ishak scores in patients with HCV-sustained virologic response [79]. However, clinical translation of BMS-986263 still faces significant challenges.

Several issues must be addressed to advance nanomedicine development. First, most nanocarriers target a single aspect of MASLD progression. For example, IR is the central pathogenesis in the progression of MASLD. Treatments focused solely on anti-inflammatory or anti-fibrotic agents do not address the full spectrum of disease progression. The complex interplay between different cell types and organs, as well as complications like hypertension, demand urgent attention. A comprehensive therapy targeting both the primary symptoms of MASLD and its complications, such as portal hypertension, may represent a future development trend. Compared to traditional therapies, nanotechnology-based drugs more readily enable multifunctional, multi-target synergistic effects for MASLD treatment. A multifunctional, one-medication-fits-all approach could be more effective for future MASLD treatments.

Additionally, most nanomedicines are still in pre-clinical stages, and their long-term toxicity, immunogenicity, pharmacokinetics, and efficacy require further clinical evaluation. Many nanodrugs have failed in clinical development due to limited improvement in efficacy [106]. Differences in etiology, pathology, and drug response between pre-clinical models and humans are also major challenges in developing nanomedicines. This highlights the need for experimental animal models that closely replicate human pathophysiological processes.

Furthermore, designing a nanocarrier with minimal toxicity and excellent tolerability is crucial. Safety guidelines specific to nanomedicines are still lacking, complicating regulatory assessments. Nanocarrier properties like absorption, distribution, metabolism, and excretion are closely related to therapeutic effects and biological activity and should be thoroughly investigated. Additionally, barriers such as LSECs capillarization, ECM accumulation, and the endothelial reticular system must be systematically considered to enhance targeted liver cell delivery. For instance, in liver-targeted delivery, the endothelial reticular system comprising Kupffer cells (KCs) and LSECs is the first barrier nanodrugs encounter. Uptake by KCs can reduce delivery to other liver sites, and loss of fenestration hinders the passage of nanodrugs from blood to hepatocytes and activated HSCs. The accumulation of ECM in the peri‑sinusoidal space can also impede the transport of NPs to other liver cells.

This review highlights the mechanisms, advancements, and challenges of nanomedicine strategies for MASLD. Continuous efforts are required to improve the diagnosis and treatment of MASLD.

## Conflicts of interest

The authors have declared no conflict of interest.
